# Porcine-derived acellular dermal matrix in primary augmentation mammoplasty to minimize implant-related complications and achieve an internal mastopexy: a case series

**DOI:** 10.1186/1752-1947-7-275

**Published:** 2013-12-30

**Authors:** Andrew Kornstein

**Affiliations:** 1 Museum Mile Surgery Center, 1050 Fifth Avenue, New York, NY 10028, USA

**Keywords:** Acellular dermal matrix, Breast augmentation, Capsular contraction, Implant malposition, Porcine-derived acellular dermal matrix, Poor soft-tissue support, Internal mastopexy, Strattice

## Abstract

**Introduction:**

Patients who present for augmentation mammoplasty with poor quality mammary soft-tissue support may be at increased risk for post-operative complications. Non-crosslinked intact porcine-derived acellular dermal matrix (Strattice™ Reconstructive Tissue Matrix, LifeCell Corp., Branchburg, NJ, USA) may enhance soft-tissue support in such patients and reduce implant-related complications, including capsular contracture, rippling, palpability, and malposition. The objective of this case report series was to describe the outcomes of three patients with poor quality mammary soft-tissue support who underwent primary cosmetic breast augmentation with pre-emptive implantation of porcine-derived acellular dermal matrix.

**Case presentation:**

Case 1 concerns a 40-year-old Caucasian woman with post-partum soft tissue laxity and grade II ptosis. Case 2 concerns a 30-year-old Caucasian woman with congenital soft-tissue laxity and grade I + ptosis. Case 3 concerns a 49-year-old Caucasian woman with post-partum and post-weight-loss-induced laxity and grade III ptosis. In all three of our patients, porcine-derived acellular dermal matrix was sutured to the chest wall along the infra-mammary and/or a neo-infra-mammary fold and then laid passively superiorly or sutured under tension to the breast parenchyma or caudal edge of the pectoralis major muscle. In cases 1 and 2, a modified internal mastopexy technique was performed. Suturing the porcine-derived acellular dermal matrix to the posterior aspect of the breast parenchyma and/or caudal pectoralis muscle under appropriate tension in conjunction with radial plication of the porcine-derived acellular dermal matrix created a snug ‘hand-in-glove’ pocket and resulted in only minimal peri-areolar scarring. Case 3 required a vertical scar mastopexy. During a mean of 18 months of follow-up, all three patients had positive outcomes and no complications (that is, infection, hematoma, seroma, rippling, malposition, or capsular contracture). The surgeon and patients were generally highly satisfied with the aesthetic outcome of the breasts.

**Conclusions:**

Pre-emptive use of porcine-derived acellular dermal matrix may be beneficial in patients with primary augmentation with poor quality mammary soft-tissue support.

## Introduction

The long-term success of any cosmetic surgery relies on the quality and characteristics of the overlying soft tissue. It follows that the ability to pre-operatively characterize candidates with poor-quality mammary soft tissue would enable treating physicians to identify patients at higher risk for implant-related skin and breast parenchymal stress and strain [1,2]. The pre-operative history should focus on potential causes of poor quality mammary soft tissue. When positively identified during the physical examination, this issue can be specifically addressed in the surgical plan. Some etiologies include pronounced weight loss and/or fluctuation [3], post-partum changes (especially with breastfeeding) [4], the effects of gravity and advancing age, and congenital soft-tissue laxity [5,6].

A physical examination is essential for identifying poor quality mammary soft tissue. In the absence of a validated method for quantification, physicians must rely on clinical observations and judgment. The supportive quality of mammary soft tissue may be evaluated based on the presence/absence of striae, breast parenchymal thickness assessed by pinch at the upper and lower poles, ease of parenchymal distraction at the lower pole and areola, ease of digital displacement of the infra-mammary fold from the underlying chest wall, and change in breast-tissue configuration in the ‘diver’s position’ (for example, with the patient bent over, the amount of displacement of glandular tissue toward the floor as well as traction on the skin) [6]. Greater parenchymal stretch, low elasticity, and tissue that is easily displaced from the chest wall, usually occurring in combination with varying degrees of ptosis, indicate poor quality soft-tissue support [6,7]. In our practice, this examination is a standard procedure for all patients considering any form of mammoplasty.

**Figure 1:**
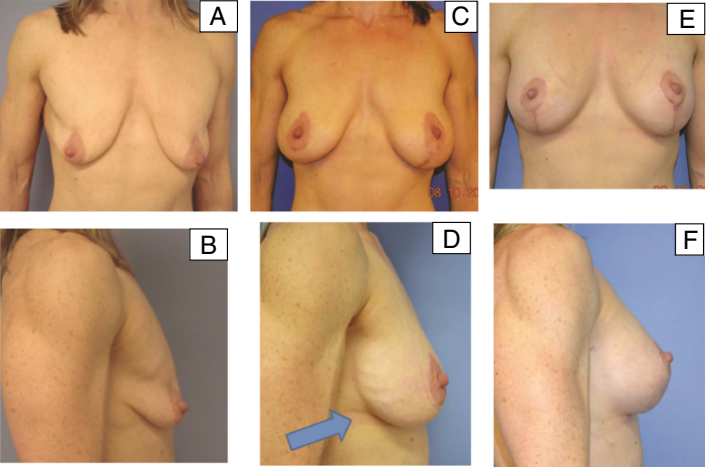
**Example of primary augmentation without the use of porcine-derived acellular dermal matrix.** Our patient (a 45-year-old woman with poor mammary soft-tissue support) underwent bilateral augmentation with left-sided mastopexy without the use of porcine-derived acellular dermal matrix. **(A,B)** Pre-operative frontal views show glandular ptosis marked by separation between the chest wall soft tissues. **(C,D)** Early post-operative views after initial surgery show glandular ptosis and separation between the chest wall soft tissues to still be present, as well as descending breast implant and stress relaxation of mammary soft tissues under the influence of implant weight without porcine-derived acellular dermal matrix. In the lateral view, the arrow shows rippling on the lateral breast surface. Because of poor lower pole support, soon after her primary surgery, our patient was deemed a candidate for bilateral revisional augmentation with porcine-derived acellular dermal matrix for inferior pole support. **(E)** The frontal view shows improved implant lift and upper breast fullness after bilateral revisional augmentation with porcine-derived acellular dermal matrix. **(F)** The lateral view shows resolution of rippling.

As noted previously by others [6-8] and in agreement with our experience, patients with these characteristics are at increased risk for complications following mammoplasty (augmentation or mastopexy), such as capsular contracture, implant malposition, rippling, palpability, and recurrent ‘glandular’ ptosis. Figure 1 shows this phenomenon prior to breast augmentation (Figure 1A,B) and early in the post-operative course (Figure 1C,D). Others [9,10] have previously noted that increased stress or tension on soft tissue, as with a poorly supported breast implant, may contribute to a higher risk for fibrotic scar tissue formation, similar to what is seen with capsular contracture. In this way, poor quality soft-tissue support potentially plays a contributory role, in conjunction with other factors known to increase capsular contracture risk, such as subglandular implant position, smooth surfaced implants, and bacterial colonization of the peri-prosthetic space and/or implant surface [11-13]. A reliable, safe, and reproducible means of pre-emptively restoring architectural structural stability to poor quality mammary soft tissue may thus help to reduce this and other implant-related complications and their associated costs both in terms of time and expense. In turn, reducing complications might improve patient and surgeon satisfaction with long-term cosmetic breast augmentation outcomes.

Acellular dermal matrices (ADMs) have been used widely in prosthetic breast reconstruction [14-16] and in revision of cosmetic breast surgery [17,18]. Among the most widely used of these is a non-crosslinked intact porcine-derived ADM (PADM; Strattice™ Reconstructive Tissue Matrix, LifeCell Corp., Branchburg, NJ, USA), which serves as a scaffold for the ingrowth of host cells, collagen, and blood vessels. The use of ADMs has been relatively more limited for cosmetic breast procedures, likely due in part to direct product cost. Moreover, some investigators have reported an increased risk of certain complications with ADMs in the context of breast reconstruction, including infection and reconstruction failure, but a lower rate of capsular contracture; however, it is not yet known whether different ADMs pose different levels of risk [19]. Regardless, to date only a small number of published reports have described the successful use of ADMs in primary cosmetic breast procedures to create ‘internal bras’ in patients with skin of poor quality or evidence of ligamentous support failure undergoing mastopexy, either alone or paired with breast reduction [8,14]. By contrast, women with poor quality soft-tissue support seeking breast augmentation are widely considered at increased risk for a poor long-term outcome [8]. Consequently, cosmetic procedure options for such patients have been limited [8].

A positive four-year experience in revisional aesthetic breast surgery cases where PADM was used to provide additional soft-tissue support led us to consider the possible value of pre-emptive use of PADM in primary augmentation procedures in patients identified pre-operatively as having similar soft-tissue characteristics. In our opinion, this approach, in appropriately selected patients, could potentially prevent or reduce the risk of complications and issues associated with revisional procedures, including out-of-pocket patient costs.

This case report series describes surgical procedures and outcomes in the first three patients with poor quality mammary soft tissue who underwent primary cosmetic breast implant surgery since November 2011. All three of our patients were identified pre-operatively as being at increased risk of post-operative soft-tissue complications, based on history and physical examination using techniques described by Tebbetts to characterize poor quality mammary soft-tissue support [6]. PADM was incorporated pre-emptively into the procedure to minimize untoward cosmetic sequelae (for example, capsular contracture, rippling, and implant malposition, and the need for re-operation) as well as the expense and loss of productivity associated with revisional surgery. A new technique of internal mastopexy was also used to limit unsightly scarring of the breast.

## Case presentation

### Patient 1

**Figure 2:**
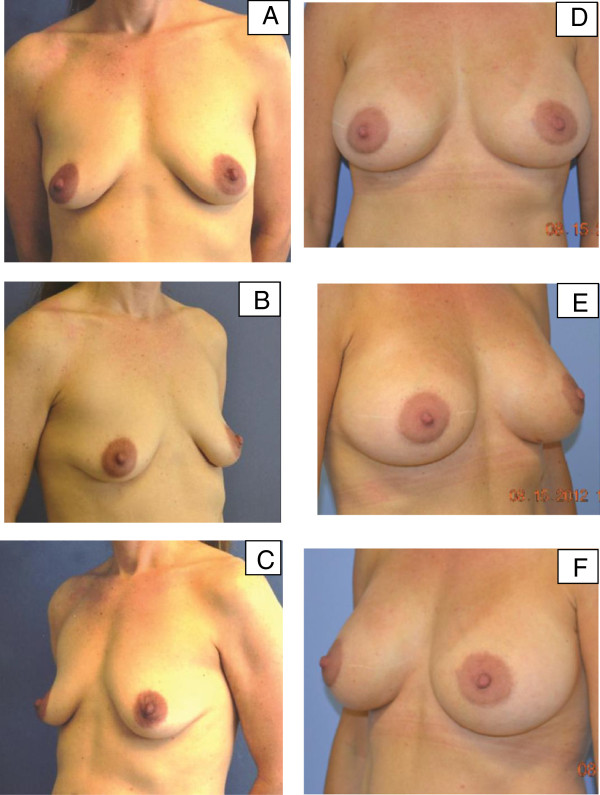
**Pre-operative and post-operative images for patient 1. (A-C)** Pre-operative images for our first patient, a 40-year-old woman with poor quality mammary soft-tissue support due to post-partum changes. **(D-F)** Six-month post-operative images following bilateral peri-areolar augmentation with porcine-derived acellular dermal matrix.

A 40-year-old Caucasian woman was evaluated at our facility for primary cosmetic breast implant surgery. Our patient was 1.55m tall, weighed 52.2kg and had poor quality mammary soft-tissue support related to post-partum involution, a history of significant gestational weight gain, and breastfeeding. Post-partum changes were accompanied by grade II ptosis (Figure 2A-C). Her medical history was unremarkable, with two pregnancies, absence of obesity or smoking, no report of major weight loss, and no personal or family history of breast cancer. During her initial consultation, our patient expressed a desire for implants that were very large relative to her small frame and limited amount of soft-tissue support. The potential rewards and risks of the large implant size were carefully and meticulously discussed with our patient and her husband prior to surgery; potential future issues relating to the breast as well as neck and back pain were addressed. In our patient’s case, consent to use PADM was mandatory in order to proceed with the surgery.

**Figure 3:**
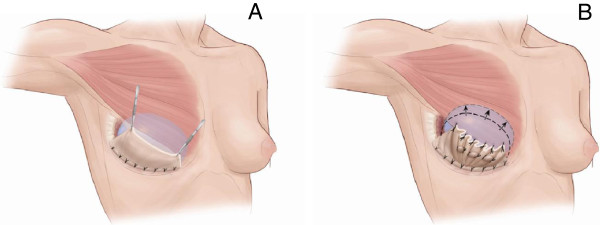
**Porcine-derived acellular dermal matrix suturing technique. (A)** Artist’s rendering of the porcine-derived acellular dermal matrix sutured to the infra-mammary fold and draped over the implant with the superior edge approximating the inferolateral margin of the pectoralis major. **(B)** Suture plication of the porcine-derived acellular dermal matrix to help maintain a higher position of the implant on the chest wall. This includes plication of the porcine-derived acellular dermal matrix as needed, not only at the periphery but within the central areas. Reproduced with permission from Rob Flewell (Certified Medical Illustration, Mebane, NC, USA).

Our patient underwent bilateral peri-areolar augmentation with PADM used for inferior pole support. Antibiotic agents were used intra-operatively and post-operatively as prophylaxis against wound infection and for the prevention and/or elimination of biofilms and breast pocket colonization associated with capsular contracture. Specifically, vancomycin 500mg was administered for peri-operative prophylaxis, followed by moxifloxacin 400mg/day for seven days; these agents were selected, in part, based on evidence that they prevent and/or eliminate biofilms [20,21]. Intra-operatively, the subpectoral surgical pockets and implants were irrigated with triple antibiotic solution (cefazolin 1g/gentamycin 80mg/bacitracin 50,000U in 500mL of normal saline (Adam’s solution)) [22,23]. After general anesthesia, breast augmentation was performed through a peri-areolar incision. For each breast, one sheet (10 × 16 × 2) of PADM was irrigated with Adam’s solution and implanted along the infra-mammary fold and secured using 4-0 Mersilene® sutures (Ethicon, Somerville, NJ, USA). Both breasts received 492cc smooth round silicone implants (Allergan, Irvine, CA, USA). Plication of the PADM implant was undertaken with sutures placed 2 to 3cm apart to reduce the radius of the pocket and modify the height and position of the implant, thus achieving a modified internal mastopexy while minimizing scarring (Figure 3A,B). For each breast, Jackson-Pratt round 7 FR drains were placed via the axilla between the PADM and the breast tissue. The breast parenchyma was closed with 4-0 Vicryl® sutures (Ethicon) and the areolar skin was closed in layers with 4-0 and 5-0 Monocryl® (Ethicon) sutures.

Our patient did well post-operatively. Pain and inflammation were managed pre-emptively with celecoxib 200mg daily starting two days before surgery [24]. Post-operatively, analgesia regimens included acetaminophen (500mg) cyclobenzaprine (10mg), and hydrocodone/acetaminophen (dose variable). Our patient returned for follow-up five days post-operatively for surgical dressing removal. Drains were removed once the drainage was <25cc/24 hours for two consecutive days. Figure 2D-F shows the results at six months after surgery. Post-operative recovery was uneventful and no complications occurred. At 16 months after the procedure, our patient and surgeon were both pleased with the aesthetic outcome.

### Patient 2

**Figure 4:**
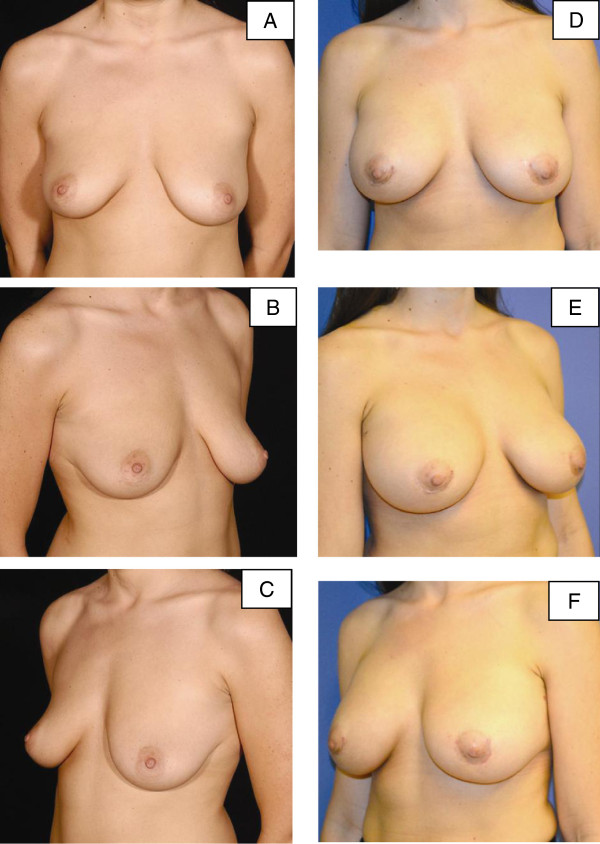
**Pre-operative and post-operative images for patient 2. (A-C)** Pre-operative images for our second patient, a 30-year-old woman with congenital soft-tissue laxity. **(D-F)** Eight-month post-operative images following peri-areolar augmentation with lower pole non-crosslinked intact porcine-derived acellular dermal matrix. As with patient 1, radial plication of the porcine-derived acellular dermal matrix obviated the need for mastopexy.

A 30-year-old Caucasian woman with congenital soft-tissue laxity, based on physical examination and history devoid of known risk factors, presented for primary cosmetic breast augmentation (Figure 4A-C). Our patient was 1.63m tall, weighed 65.3kg, and had grade 1+ ptosis. Her medical history was marked by clinically significant weight loss (10% of previous maximum weight 72.3kg), absence of obesity or smoking, and no personal or family history of breast cancer. During her initial consultation, our patient expressed a wish for minimal scarring. Our patient underwent bilateral peri-areolar augmentation with inferior pole PADM placement. Peri-operative management and intra-operative technique were as described for patient 1. For each breast, one sheet (10 × 16 × 2) of PADM was implanted along the infra-mammary fold and secured using 3-0 Mersilene sutures. Both breasts received 330cc smooth round silicone implants (Allergan). As with patient 1, radial plication of the PADM obviated the need for mastopexy.

Her post-operative course was unremarkable. Figure 4 D-F shows results at eight months after surgery. Although the nipple areolar complex position is slightly lower than ideal, the overall outcome, in light of her small areola size, virginal breast mound, and more global body laxity, is aesthetically pleasing. Our patient is also pleased that, with this result, there is no vertical scar and only a barely visible peri-areolar scar. Our patient had a high level of satisfaction with the aesthetic outcome at 18 months after surgery.

### Patient 3

**Figure 5:**
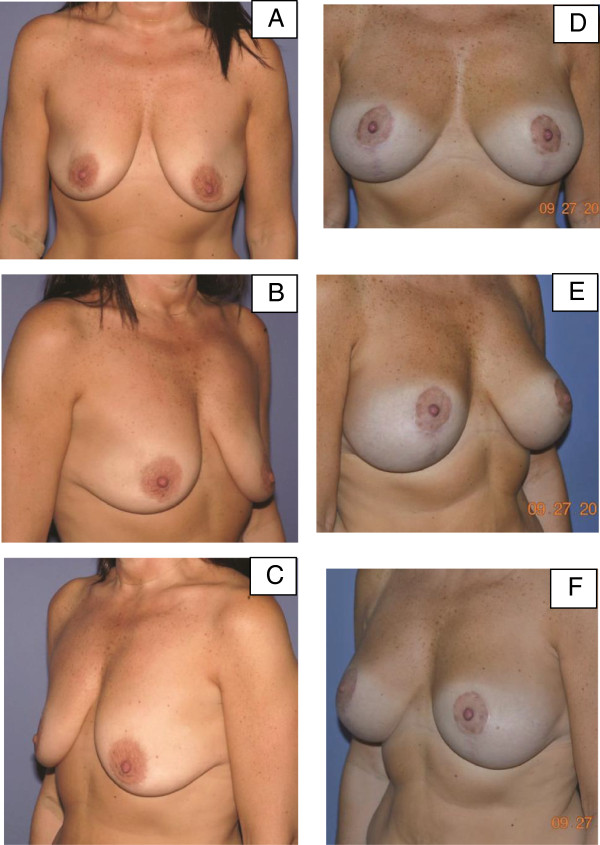
**Pre-operative and post-operative images for patient 3. (A-C)** Pre-operative images for our third patient, a 49-year-old woman who had poor quality mammary soft-tissue support due to weight loss, pregnancy, and breastfeeding. **(D-F)** Post-operative images at 12 months following bilateral augmentation and vertical scar mastopexy with porcine-derived acellular dermal matrix.

A 49-year-old Caucasian woman was evaluated for primary cosmetic breast augmentation (Figure 5A-C). Our patient was 1.57m tall, weighed 65.8kg, and had grade III ptosis. She was a non-smoker who had undergone two pregnancies (maximum weight while pregnant, 70.3kg). Our patient had poor quality mammary soft-tissue support based on her physical examination and history, which included pregnancy and significant gestational weight gain, breastfeeding, post-partum involution, and subsequent fluctuating weight gain and weight loss. There was no family history of breast cancer. Our patient underwent bilateral augmentation and vertical scar mastopexy with two 5 × 16 × 1cm sheets of PADM implanted along the infra-mammary fold of each breast and secured with 2-0 Mersilene sutures. Peri-operative management and intra-operative technique were otherwise as described for patient 1. Both breasts received 371cc smooth round silicone implants (Allergan). Figure 5D-F shows results at 12 months. No post-operative complications occurred in our patient. At 21 months after surgery, she was very happy with the results.

## Discussion

All three patients who underwent primary cosmetic breast augmentation with pre-emptive use of PADM in the setting of poor mammary soft-tissue quality had positive outcomes. There were no reports of post-operative infection, hematoma, seroma, recurrent ptosis, breast pain, loss of nipple sensation, rippling, or capsular contracture during post-operative follow-up, ranging from 16 to 21 months (mean, 18 months). All our patients were highly satisfied with their aesthetic outcome. The surgeon was also satisfied with the post-operative breast aesthetics in all three patients. Furthermore, none of our patients required revisions or any of the attendant issues (time, expense) associated with other procedures to correct complications.

The outcomes detailed in these three patients are in line with our clinical experience as well as that of others [6-8], which suggested that poor quality mammary soft-tissue support may be a potential harbinger of untoward complications in patients with cosmetic breast implants. Among the most commonly encountered complications and adverse outcomes experienced by patients with breast implants in general are capsular contractures, reported to occur at rates of 8.1% at three years and 20.5% at six years of follow-up in large prospective US Food and Drug Administration (FDA)-mandated manufacturers’ studies [25,26]. Of note, most (92%) capsular contractures are reported within the first 12 months after surgery [27].

A number of theories exist regarding the etiology of capsular contracture. Infection and hematoma are commonly implicated as causative factors [13,28]. In addition to infectious and hematoma-related etiologies for capsular contracture, it is our hypothesis based on clinical experience that risk for capsular contracture may be heightened when the breast parenchyma’s support systems are compromised or weakened. This is in line with evidence showing that parenchymal or dermal tension, compression, or stretching are associated with increased scar tissue formation [9,10]. Other variables associated with an elevated risk of capsular contracture include a smooth implant surface [11] and subglandular implant positioning [29]. Furthermore, bacterial colonization of the peri-prosthetic space is associated with Baker grade III or IV capsular contracture [12,13].

In addition to the potentially positive impact of PADM use in our current patients, the consistent intra-operative use of a vancomycin, moxifloxacin, and triple-antibiotic solution for irrigation may have contributed to prevention of capsular contracture and other contamination-related complications. Bacteria introduced into the implant pocket at the time of surgery have been hypothesized to form an antibiotic-resistant biofilm that adheres to the breast implant surface, which may lead to chronic inflammation and growth of fibrous tissue over time [12,13]. Fortunately, the importance of using an antibiotic irrigation solution has become more widely recognized among many plastic surgeons who perform breast reconstruction and cosmetic procedures [22,23]. As new cohorts of patients with breast procedures are followed, it will be of interest to examine the impact of antibacterial irrigation techniques on capsular contracture rates. At the present time, the risk of capsular contracture appears to be multi-factorial and all potentially causative agents should be addressed. Poor soft-tissue mammary support may be at least partially responsible for apparent spontaneous capsular contracture occurring remotely from the date of surgery.

It is important to note that the current findings are based on a series of only three patients and are observational in nature; thus, a causative association between use of PADM and prevention of capsular contracture in patients with primary breast augmentation cannot be established. Future controlled investigations are needed to confirm whether this is indeed the case.

It is notable that all three cases described here are representative of a class of patient with cosmetic breast augmentation that is typically regarded as increased risk (women with poor quality soft-tissue support seeking breast augmentation) and limited in their cosmetic treatment options. The surgical method described is a potentially novel use of PADM that requires further investigation. As with any cosmetic elective procedure, the costs and risk to benefit ratio must be carefully weighed in such cases. The current surgical use of PADM is similar to that described in a recent report of the successful use of ADM as an internal bra for patients with ptosis undergoing primary mastopexy, either alone or in combination with breast reduction [8]. The current findings supplement those of this earlier study and suggest that PADM also may be used in similar patients who wish to undergo breast implant surgery. ADMs are likely to be of particular value in such patients because they provide a connective tissue matrix to reinforce weakened tissues, limit tension on the skin envelope, and successfully support an implant [14,23,30].

## Conclusions

In three patients with poor quality mammary soft-tissue support due to a variety of factors, the pre-emptive use of PADM during primary breast augmentation may have contributed to positive outcomes, marked by minimal complications and high patient satisfaction. Additional follow-up of these and similar patients is planned, and will be reported to assess long-term outcomes.

## Consent

Written informed consent was obtained from all three patients for publication of this case report and accompanying images. Copies of the written consents are available for review by the Editor-in-Chief of this journal.

## Abbreviations

ADMs: Acellular dermal matrices; PADM: Porcine-derived acellular dermal matrix.

## Competing interests

The author declares that he has no competing interests.

## Authors’ contributions

AK performed the surgeries for all patients in the case study series, performed all follow-up assessments, was a major contributor in writing the manuscript, and approved the final manuscript for submission.
